# Prediction of Breast and Prostate Cancer Risks in Male *BRCA1* and *BRCA2* Mutation Carriers Using Polygenic Risk Scores

**DOI:** 10.1200/JCO.2016.69.4935

**Published:** 2017-04-27

**Authors:** Julie Lecarpentier, Valentina Silvestri, Karoline B. Kuchenbaecker, Daniel Barrowdale, Joe Dennis, Lesley McGuffog, Penny Soucy, Goska Leslie, Piera Rizzolo, Anna Sara Navazio, Virginia Valentini, Veronica Zelli, Andrew Lee, Ali Amin Al Olama, Jonathan P. Tyrer, Melissa Southey, Esther M. John, Thomas A. Conner, David E. Goldgar, Saundra S. Buys, Ramunas Janavicius, Linda Steele, Yuan Chun Ding, Susan L. Neuhausen, Thomas V.O. Hansen, Ana Osorio, Jeffrey N. Weitzel, Angela Toss, Veronica Medici, Laura Cortesi, Ines Zanna, Domenico Palli, Paolo Radice, Siranoush Manoukian, Bernard Peissel, Jacopo Azzollini, Alessandra Viel, Giulia Cini, Giuseppe Damante, Stefania Tommasi, Paolo Peterlongo, Florentia Fostira, Ute Hamann, D. Gareth Evans, Alex Henderson, Carole Brewer, Diana Eccles, Jackie Cook, Kai-ren Ong, Lisa Walker, Lucy E. Side, Mary E. Porteous, Rosemarie Davidson, Shirley Hodgson, Debra Frost, Julian Adlard, Louise Izatt, Ros Eeles, Steve Ellis, Marc Tischkowitz, EMBRACE, Andrew K. Godwin, Alfons Meindl, Andrea Gehrig, Bernd Dworniczak, Christian Sutter, Christoph Engel, Dieter Niederacher, Doris Steinemann, Eric Hahnen, Jan Hauke, Kerstin Rhiem, Karin Kast, Norbert Arnold, Nina Ditsch, Shan Wang-Gohrke, Barbara Wappenschmidt, Dorothea Wand, Christine Lasset, Dominique Stoppa-Lyonnet, Muriel Belotti, Francesca Damiola, Laure Barjhoux, Sylvie Mazoyer, GEMO Study Collaborators, Mattias Van Heetvelde, Bruce Poppe, Kim De Leeneer, Kathleen B.M. Claes, Miguel de la Hoya, Vanesa Garcia-Barberan, Trinidad Caldes, Pedro Perez Segura, Johanna I. Kiiski, Kristiina Aittomäki, Sofia Khan, Heli Nevanlinna, Christi J. van Asperen, HEBON, Tibor Vaszko, Miklos Kasler, Edith Olah, Judith Balmaña, Sara Gutiérrez-Enríquez, Orland Diez, Alex Teulé, Angel Izquierdo, Esther Darder, Joan Brunet, Jesús Del Valle, Lidia Feliubadalo, Miquel Angel Pujana, Conxi Lazaro, Adalgeir Arason, Bjarni A. Agnarsson, Oskar Th. Johannsson, Rosa B. Barkardottir, Elisa Alducci, Silvia Tognazzo, Marco Montagna, Manuel R. Teixeira, Pedro Pinto, Amanda B. Spurdle, Helene Holland, KConFab Investigators, Jong Won Lee, Min Hyuk Lee, Jihyoun Lee, Sung-Won Kim, Eunyoung Kang, Zisun Kim, Priyanka Sharma, Timothy R. Rebbeck, Joseph Vijai, Mark Robson, Anne Lincoln, Jacob Musinsky, Pragna Gaddam, Yen Y. Tan, Andreas Berger, Christian F. Singer, Jennifer T. Loud, Mark H. Greene, Anna Marie Mulligan, Gord Glendon, Irene L. Andrulis, Amanda Ewart Toland, Leigha Senter, Anders Bojesen, Henriette Roed Nielsen, Anne-Bine Skytte, Lone Sunde, Uffe Birk Jensen, Inge Sokilde Pedersen, Lotte Krogh, Torben A. Kruse, Maria A. Caligo, Sook-Yee Yoon, Soo-Hwang Teo, Anna von Wachenfeldt, Dezheng Huo, Sarah M. Nielsen, Olufunmilayo I. Olopade, Katherine L. Nathanson, Susan M. Domchek, Christa Lorenchick, Rachel C. Jankowitz, Ian Campbell, Paul James, Gillian Mitchell, Nick Orr, Sue Kyung Park, Mads Thomassen, Kenneth Offit, Fergus J. Couch, Jacques Simard, Douglas F. Easton, Georgia Chenevix-Trench, Rita K. Schmutzler, Antonis C. Antoniou, Laura Ottini

**Affiliations:** Julie Lecarpentier, Karoline B. Kuchenbaecker, Daniel Barrowdale, Joe Dennis, Lesley McGuffog, Goska Leslie, Andrew Lee, Ali Amin Al Olama, Jonathan P. Tyrer, Debra Frost, Steve Ellis, Douglas F. Easton, and Antonis C. Antoniou, University of Cambridge; Karoline B. Kuchenbaecker, The Wellcome Trust Sanger Institute, Hinxton; Marc Tischkowitz, Addenbrooke’s Treatment Centre, Addenbrooke’s Hospital, Cambridge; D. Gareth Evans, Manchester University, Central Manchester University Hospitals NHS Foundation Trust, Manchester; Alex Henderson, Newcastle Upon Tyne Hospitals NHS Trust, Newcastle upon Tyne; Carole Brewer, Royal Devon and Exeter Hospital, Exeter; Diana Eccles, Southampton University Hospitals NHS Trust, Southampton; Jackie Cook, Sheffield Children’s Hospital, Sheffield; Kai-ren Ong, Birmingham Women’s Hospital Healthcare NHS Trust, Edgbaston, Birmingham; Lisa Walker, Churchill Hospital, Oxford; Lucy E. Side, Great Ormond Street Hospital for Children NHS Trust; Shirley Hodgson, St George's, University of London; Louise Izatt, Guy’s and St Thomas’ NHS Foundation Trust; Ros Eeles, The Institute of Cancer Research and Royal Marsden NHS Foundation Trust; Nick Orr, The Institute of Cancer Research, London; Mary E. Porteous, Western General Hospital, Edinburgh; Rosemarie Davidson, South Glasgow University Hospitals, Glasgow; Julian Adlard, Chapel Allerton Hospital, Leeds, United Kingdom; Valentina Silvestri, Piera Rizzolo, Anna Sara Navazio, Virginia Valentini, Veronica Zelli, and Laura Ottini, Sapienza University of Rome, Rome; Angela Toss, Veronica Medici, and Laura Cortesi, University of Modena and Reggio Emilia, Modena; Ines Zanna and Domenico Palli, Cancer Research and Prevention Institute, Florence; Paolo Radice, Siranoush Manoukian, Bernard Peissel, and Jacopo Azzollini, Fondazione Istituto Di Ricovero e Cura a Carattere Scientifico (IRCCS) Istituto Nazionale Tumori (INT); Paolo Peterlongo, Italian Foundation for Cancer Research Institute of Molecular Oncology (IFOM), Milan; Alessandra Viel and Giulia Cini, CRO Aviano, National Cancer Institute, Aviano; Giuseppe Damante, University of Udine, Udine; Stefania Tommasi, Istituto Nazionale Tumori “Giovanni Paolo II”, Bari; Elisa Alducci, Silvia Tognazzo, and Marco Montagna, Veneto Institute of Oncology IOV - IRCCS, Padua; Maria A. Caligo, University and University Hospital of Pisa, Pisa, Italy; Penny Soucy and Jacques Simard, Centre Hospitalier Universitaire de Québec Research Center and Laval University, Quebec City, Quebec; Anna Marie Mulligan and Irene L. Andrulis, University of Toronto; Gord Glendon and Irene L. Andrulis, Mount Sinai Hospital, Toronto, Ontario, Canada; Melissa Southey, Ian Campbell, Paul James, and Gillian Mitchell, University of Melbourne, Parkville, Victoria; Amanda B. Spurdle, Helene Holland, and Georgia Chenevix-Trench, QIMR Berghofer Medical Research Institute, Brisbane, Queensland; Ian Campbell, Paul James, and Gillian Mitchell, Peter MacCallum Cancer Centre, East Melbourne, New South Wales, Australia; Esther M. John, Cancer Prevention Institute of California, Fremont; Linda Steele, Yuan Chun Ding, Susan L. Neuhausen, and Jeffrey N. Weitzel, City of Hope, Duarte, CA; Thomas A. Conner and Saundra S. Buys, Huntsman Cancer Institute; David E. Goldgar, University of Utah School of Medicine, Salt Lake City, UT; Andrew K. Godwin, University of Kansas Medical Center, Kansas City; Priyanka Sharma, University of Kansas Medical Center, Westwood, KS; Timothy R. Rebbeck, Harvard TH Chan School of Public Health and Dana Farber Cancer Institute, Boston, MA; Joseph Vijai, Mark Robson, Anne Lincoln, Jacob Musinsky, Pragna Gaddam, and Kenneth Offit, Memorial Sloan Kettering Cancer Center, New York, NY; Jennifer T. Loud and Mark H. Greene, National Cancer Institute, Bethesda, MD; Amanda Ewart Toland and Leigha Senter, The Ohio State University, Columbus, OH; Dezheng Huo, Sarah M. Nielsen, and Olufunmilayo I. Olopade, University of Chicago Medical Center, Chicago, IL; Katherine L. Nathanson and Susan M. Domchek, University of Pennsylvania, Philadelphia; Christa Lorenchick and Rachel C. Jankowitz, University of Pittsburgh Medical Center, Pittsburgh, PA; Fergus J. Couch, Mayo Clinic, Rochester, MN; Ramunas Janavicius, State Research Institute Innovative Medicine Center, Vilnius, Lithuania; Thomas V.O. Hansen, Rigshospitalet, Copenhagen University Hospital, Copenhagen; Anders Bojesen and Henriette Roed Nielsen, Vejle Hospital, Vejle; Anne-Bine Skytte, Lone Sunde, and Uffe Birk Jensen, Aarhus University Hospital, Aarhus; Inge Sokilde Pedersen, Aalborg University Hospital, Aalborg; Lotte Krogh, Torben A. Kruse, and Mads Thomassen, Odense University Hospital, Odense, Denmark; Ana Osorio, National Cancer Research Centre and Spanish Network on Rare Diseases; Miguel de la Hoya, Vanesa Garcia-Barberan, Trinidad Caldes, and Pedro Perez Segura, Hospital Clinico San Carlos, El Instituto de Investigación Sanitaria del Hospital Clínico San Carlos, Madrid; Judith Balmaña, University Hospital, Vall d'Hebron; Sara Gutiérrez-Enríquez and Orland Diez, Vall d’Hebron Institute of Oncology; Orland Diez, University Hospital Vall d’Hebron; Alex Teulé, Jesús Del Valle, Lidia Feliubadalo, Miquel Angel Pujana, and Conxi Lazaro, Bellvitge Biomedical Research Institute, Catalan Institute of Oncology, Barcelona; Angel Izquierdo, Esther Darder, and Joan Brunet, Institut d'Investigació Biomèdica de Girona, Catalan Institute of Oncology, Girona, Spain; Florentia Fostira, National Centre for Scientific Research “Demokritos,” Athens, Greece; Ute Hamann, German Cancer Research Center (DKFZ); Christian Sutter, University Hospital Heidelberg, Heidelberg; Alfons Meindl, Klinikumrechts der Isar, Technical University Munich; Nina Ditsch, Ludwig-Maximilian University, Munich; Andrea Gehrig, University Würzburg, Würzburg; Bernd Dworniczak, University of Münster, Münster; Christoph Engel, University of Leipzig; Dorothea Wand, University Hospital, Leipzig; Dieter Niederacher, University Hospital Düsseldorf, Heinrich-Heine University, Düsseldorf; Doris Steinemann, Hannover Medical School, Hannover; Eric Hahnen, Jan Hauke, Kerstin Rhiem, Barbara Wappenschmidt, and Rita K. Schmutzler, University Hospital Cologne, Cologne; Karin Kast, University Hospital Carl Gustav Carus, Technical University Dresden, Dresden; Norbert Arnold, University Hospital of Schleswig-Holstein, Christian-Albrechts University Kiel, Kiel; Shan Wang-Gohrke, University Hospital Ulm, Ulm, Germany; Christine Lasset, Francesca Damiola, and Laure Barjhoux, Centre Léon Bérard; Sylvie Mazoyer, University of Lyon, Lyon; Dominique Stoppa-Lyonnet and Muriel Belotti, Institut Curie, Paris, France; Mattias Van Heetvelde, Bruce Poppe, Kim De Leeneer, and Kathleen B.M. Claes, Ghent University, Gent, Belgium; Johanna I. Kiiski, Sofia Khan, and Heli Nevanlinna, University of Helsinki; Johanna I. Kiiski, Kristiina Aittomäki, Sofia Khan, and Heli Nevanlinna, Helsinki University Hospital, Helsinki, Finland; Christi J. van Asperen, Leiden University Medical Center, Leiden, the Netherlands; Tibor Vaszko, Miklos Kasler, and Edith Olah, National Institute of Oncology, Budapest, Hungary; Adalgeir Arason, Bjarni A. Agnarsson, Oskar Th. Johannsson, and Rosa B. Barkardottir, Landspitali University Hospital and Biomedical Centre, University of Iceland, Reykjavik, Iceland; Manuel R. Teixeira and Pedro Pinto, Portuguese Oncology Institute; Manuel R. Teixeira, Porto University, Porto, Portugal; Jong Won Lee, Ulsan College of Medicine and Asan Medical Center; Min Hyuk Lee and Jihyoun Lee, Soonchunhyang University and Hospital; Sung-Won Kim and Eunyoung Kang, Daerim St Mary’s Hospital; Sue Kyung Park, Seoul National University College of Medicine, Seoul; Zisun Kim, Soonchunhyang University Bucheon Hospital, Bucheon, Korea; Yen Y. Tan, Andreas Berger, and Christian F. Singer, Medical University of Vienna, Vienna, Austria; Sook-Yee Yoon and Soo-Hwang Teo, Sime Darby Medical Centre, Subang Jaya, Malaysia; and Anna von Wachenfeldt, Karolinska University Hospital, Stockholm, Sweden.

## Abstract

**Purpose:**

*BRCA1/2* mutations increase the risk of breast and prostate cancer in men. Common genetic variants modify cancer risks for female carriers of *BRCA1/2* mutations. We investigated—for the first time to our knowledge—associations of common genetic variants with breast and prostate cancer risks for male carriers of *BRCA1*/*2* mutations and implications for cancer risk prediction.

**Materials and Methods:**

We genotyped 1,802 male carriers of *BRCA1/2* mutations from the Consortium of Investigators of Modifiers of *BRCA1/2* by using the custom Illumina OncoArray. We investigated the combined effects of established breast and prostate cancer susceptibility variants on cancer risks for male carriers of *BRCA1/2* mutations by constructing weighted polygenic risk scores (PRSs) using published effect estimates as weights.

**Results:**

In male carriers of *BRCA1/2* mutations, PRS that was based on 88 female breast cancer susceptibility variants was associated with breast cancer risk (odds ratio per standard deviation of PRS, 1.36; 95% CI, 1.19 to 1.56; *P* = 8.6 × 10^−6^). Similarly, PRS that was based on 103 prostate cancer susceptibility variants was associated with prostate cancer risk (odds ratio per SD of PRS, 1.56; 95% CI, 1.35 to 1.81; *P* = 3.2 × 10^−9^). Large differences in absolute cancer risks were observed at the extremes of the PRS distribution. For example, prostate cancer risk by age 80 years at the 5th and 95th percentiles of the PRS varies from 7% to 26% for carriers of *BRCA1* mutations and from 19% to 61% for carriers of *BRCA2* mutations, respectively.

**Conclusion:**

PRSs may provide informative cancer risk stratification for male carriers of *BRCA1/2* mutations that might enable these men and their physicians to make informed decisions on the type and timing of breast and prostate cancer risk management.

## INTRODUCTION

Germline mutations in *BRCA1* and, predominantly, *BRCA2* are associated with increased risks in men of developing breast and prostate cancers.^1,2^ *BRCA1/2* mutations account for approximately 10% of male breast cancer and 2% of prostate cancer cases.^3-5^ Breast cancer in men is rare and accounts for less than 1% of all male tumors. By contrast, prostate cancer is the most common cancer in men, accounting for approximately 25% of male tumors.^6^ The lifetime risk of male breast cancer in mutation carriers has been estimated to be 5% to 10% and 1% to 5% for carriers of *BRCA2* and *BRCA1* mutations, respectively, whereas estimates of lifetime prostate cancer risk are approximately 20% and 40% for carriers of *BRCA1* and *BRCA2* mutations, respectively.^3,7-10^

More than 100 common genetic variants (single nucleotide polymorphisms [SNPs]) that are associated with prostate cancer and female breast cancer have been identified via genome-wide association studies (GWAS) in the general population,^11,12^ and their combined effects have been shown to have significant implications for risk stratification and targeted prevention.^13-15^ By contrast, only two male breast cancer susceptibility SNPs have been identified to date,^16^ but there is some evidence that suggests that common variants that are associated with female breast cancer may influence male breast cancer risk.^17-19^

Studies by the Consortium of Investigators of Modifiers of *BRCA1/2* (CIMBA) have shown that common SNPs modify the risk of breast and ovarian cancers for female *BRCA1* and *BRCA2* mutation carriers^20-22^; however, no study to date has investigated the associations of common SNPs with breast or prostate cancer risk for men with *BRCA1*/*2* mutations and their implications for cancer risk prediction.

In this study, we performed the first GWAS for breast and prostate cancers in male *BRCA1*/2 mutation carriers enrolled in CIMBA using the custom Illumina OncoArray. Furthermore, we evaluated the combined effects of known common breast and prostate cancer susceptibility variants on cancer risks for male carriers of *BRCA1/2* mutations and estimated absolute age-specific cumulative risks of developing breast and prostate cancers on the basis of combined SNP distributions. We demonstrate—to our knowledge for the first time—that combined SNP effects have important implications for risk profiling of male carriers of *BRCA1/2* mutations.

## MATERIALS AND METHODS

### Samples

CIMBA collects data on men with *BRCA1* or *BRCA2* clearly pathogenic variants—commonly termed mutations—who are older than 18 years, with the majority recruited via cancer genetics clinics.^23^ Pathogenic variants were defined as previously described.^24^ All participating studies have been approved by local ethical review committees.

To select samples for genotyping, we used a case-control study design, selecting all available male carriers of *BRCA1/2* mutations who were affected with breast and/or prostate cancer (cases) and matching them with up to three unaffected mutation carriers (controls). Cases and controls were matched for study group or country of residence, year of birth, and gene (*BRCA1* or *BRCA2*). A total of 1,989 male carriers were selected for genotyping: 265 with breast cancer, 212 with prostate cancer, 43 with both diseases, and 1,469 unaffected.

### Genotyping and Quality Control

Genotyping was performed by using the Illumina OncoArray beadchip (approximately 570,000 SNPs with genome-wide coverage). Genotyping and quality control were performed as described in the Data Supplement. Of 1,989 samples, 1,802 passed the quality control step. We imputed genotypes using the 1000 Genomes Project as the reference panel (Data Supplement).

### Statistical Methods

#### Association Analyses.

We evaluated associations of SNPs with risks of breast and prostate cancer simultaneously using multinomial logistic regression. The control group in this analysis was defined as the set of samples without a breast or prostate cancer diagnosis. Breast and prostate cancer cases were defined on the basis of age at diagnosis, whichever occurred first. If breast and prostate cancer occurred at the same time, individuals were treated as patients with breast cancer. Thus, of 1,802 samples, 277 were defined as patients with breast cancer, 212 as patients with prostate cancer, and 1,313 as controls. Analyses were adjusted for the first three principal components, age at breast or prostate cancer for patient-cases and age at interview for controls, and gene (*BRCA1* or *BRCA2*). A robust variance approach—clustering of family membership—was used to adjust for related individuals. Additional logistic regression analyses were carried out to assess associations separately with breast or prostate cancer risk (Data Supplement). We also performed a set of sensitivity analyses by considering patient cases with both breast and prostate cancer as a separate group in a multinomial logistic regression model (Data Supplement). Analysis was performed in R (version 3.2.3; R Foundation, Vienna, Austria) and STATA software (version 13.1; STATA, College Station, TX; Computing Resource Center, Santa Monica, CA).

#### Polygenic Risk Scores.

Assuming a log-additive model for the joint effects of SNPs, we constructed polygenic risk scores (PRSs) by summing the number of alleles across SNPs that were weighted by their estimated per-allele log-odds ratios (ORs) in published studies^11,12,22,25-32^ (Data Supplement).

PRSs were standardized to have mean 0 and variance 1 (Data Supplement). We evaluated associations with quartiles of PRS on the basis of the PRS distribution in controls. Absolute age-specific cumulative risks of developing breast or prostate cancer at different percentiles of PRS were calculated using published methods^33^ (Data Supplement).

#### Selection of SNPs Included in PRSs and Weights.

##### Breast Cancer PRSs.

We investigated three main PRSs using SNPs that were known to be associated with overall risk of breast cancer or risk of estrogen receptor (ER)–positive or –negative breast cancer from published studies that were performed in females from the general population. To construct each PRS and to avoid over-fitting, we used external log-OR estimates—for their association with risk for overall breast cancer or ER-positive or ER-negative breast cancer—from the largest association studies of the Breast Cancer Association Consortium.^12,22,28-31,34^ No data from the current study were used to construct any of the PRSs. The three PRSs were defined as follows:

1. The overall PRS includes SNPs that were associated with breast cancer risk from population-based association studies. This PRS included 88 (77 genotyped, 11 imputed) SNPs.
2. The ER-positive PRS includes SNPs that were associated with ER-positive breast cancer. This PRS included 87 (76 genotyped, 11 imputed) SNPs. Weights for each SNP were based on published log-OR estimates for ER-positive breast cancer.
3. The ER-negative PRS includes SNPs associated with ER-negative disease. This PRS included 53 (47 genotyped, six imputed) SNPs. Weights for each SNP were based on log-OR estimates for ER-negative breast cancer.

A list of SNPs and weights used in each PRS is shown in the Data Supplement. To identify the most strongly associated PRS, we have evaluated the associations of all three PRSs in the set of *BRCA1* and *BRCA2* samples combined and separately.

We also investigated two PRSs by using SNPs that were associated with breast cancer risk for female *BRCA1/2* mutation carriers (Data Supplement).

##### Prostate Cancer PRS.

Prostate cancer PRS included variants that were associated with prostate cancer at genome-wide significant level in studies of the PRACTICAL consortium.^11,14,32,35-38^ Log-OR estimates from published population-based studies were used according to the approach above.^11,32^ This PRS included 103 (71 genotyped, 32 imputed) SNPs (Data Supplement).

## RESULTS

We evaluated associations for a total of 9,530,887 SNPs in 1,802 male carriers of *BRCA1/2* mutations, including 277 patients with breast cancer, 212 patients with prostate cancer, and 1,313 controls. We investigated associations in the combined sample of *BRCA1/2* mutation carriers and separately in *BRCA2* mutation carriers. The number of *BRCA1* mutation carriers was too small to allow for separate analyses. Across the two analyses, no associations at *P* < 10^−8^ were identified. A total of 577 SNPs exhibited associations at *P* < 10^−5^. GWAS results are reported in the Data Supplement.

### Breast Cancer PRSs

Of 102 SNPs included in the breast cancer PRSs, 68 SNPs (67%) yielded OR estimates in the same direction as those that have been previously reported for females in the general population. Eleven SNPs were associated with breast cancer risk at *P* < .05 (Data Supplement). After accounting for multiple testing, there was no evidence of pairwise interactions between any two variants in the PRSs.

The three main breast cancer PRSs that were constructed on the basis of associations with female breast cancer risk were strongly associated with male breast cancer risk for both *BRCA1* and *BRCA2* mutation carriers (Table 1). The OR estimate for male breast cancer per standard deviation (SD) increase in overall PRS was estimated to be 1.36 (95% CI, 1.19 to 1.56; *P* = 8.6 × 10^−6^) in combined *BRCA1/2* carriers. Associations remained significant when *BRCA1* and *BRCA2* carriers were analyzed separately (*BRCA1*: OR, 1.49; 95% CI, 1.07 to 2.07; *P* = .019; *BRCA2*: OR, 1.36; 95% CI, 1.17 to 1.58; *P* = 7.2 × 10^−5^). Men in the 3rd and 4th quartiles were at significantly increased risk of breast cancer compared with men in the bottom quartile of the PRS (Table 1), but the numbers of carriers in individual quartiles in the *BRCA1* only analyses were too small to draw definitive conclusions.

**Table 1.**
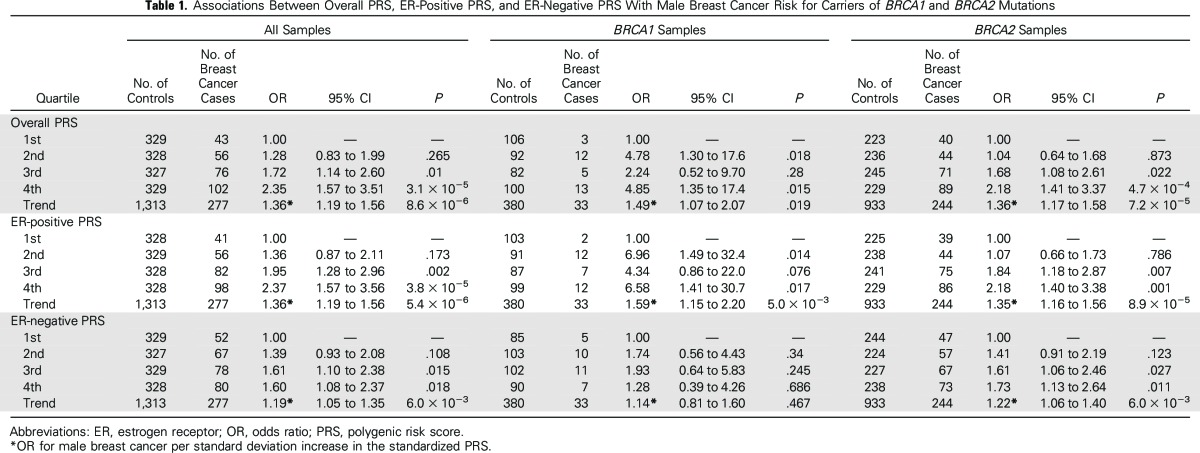
Associations Between Overall PRS, ER-Positive PRS, and ER-Negative PRS With Male Breast Cancer Risk for Carriers of *BRCA1* and *BRCA2* Mutations

The magnitude and strength of associations were similar for the PRS that was constructed on the basis of SNPs associated with ER-positive breast cancer in females (Table 1). The ER-negative PRS showed a weaker association with breast cancer risk for male carriers of *BRCA1/2* mutations. Results were similar when the associations were evaluated using logistic regression (Data Supplement) and when considering the patients with both breast and prostate cancer as a separate group in a multinomial logistic regression model (Data Supplement).

### Prostate Cancer PRS

Of 103 SNPs that were included in the prostate cancer PRS, 74 SNPs (71%) had estimated ORs in the same direction as those previously reported in population-based studies. Eight SNPs were associated at *P* < .05 (Data Supplement).

There was a highly significant association between the prostate cancer PRS and prostate cancer risk for male carriers of *BRCA1/2* mutations (OR for prostate cancer per SD increase, 1.56; 95% CI, 1.35 to 1.81; *P* = 3.2 × 10^−9^; Table 2). Associations remained significant when analyses were performed separately for carriers of *BRCA1* and *BRCA2* mutations (*BRCA1*: OR, 1.72; 95% CI, 1.30 to 2.29; *P* = 1.8 × 10^−4^; *BRCA2*: OR, 1.49; 95% CI, 1.26 to 1.77; *P* = 4.9 × 10^−6^). There was an increasing risk of prostate cancer with increasing PRS quartiles. When compared with the 1st quartile, OR for prostate cancer for men in the 2nd quartile was 1.82 (95% CI, 1.07 to 3.08; *P* = .026), for men in the 3rd quartile, 2.23 (95% CI, 1.32 to 3.76; *P* = .003), and for men in the 4th quartile, 3.36 (95% CI, 2.05 to 5.52; *P* = 1.7 × 10^−6^).

**Table 2.**
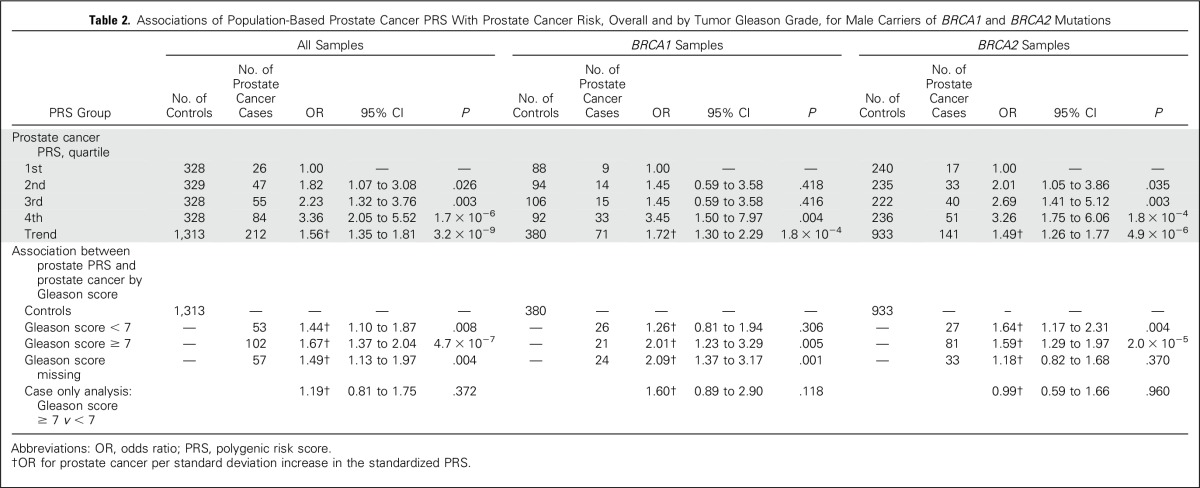
Associations of Population-Based Prostate Cancer PRS With Prostate Cancer Risk, Overall and by Tumor Gleason Grade, for Male Carriers of *BRCA1* and *BRCA2* Mutations

We observed significant associations between prostate cancer PRS with both low (< 7) and high (≥ 7) Gleason score prostate cancers (Table 2). There was no evidence of interaction between age at diagnosis and/or observation and any breast or prostate cancer PRSs (Data Supplement).

### Discriminatory Ability

The overall breast cancer and ER-positive PRSs had an area under the curve (AUC) of 0.59 (95% CI, 0.55 to 0.63). ER-negative PRS had the lowest AUC at 0.55 (95% CI, 0.51 to 0.59). The AUC for prostate cancer PRS was estimated to be 0.62 (95% CI, 0.58 to 0.66).

### Predicted Risks of Male Breast and Prostate Cancer by PRS Percentile

**Fig 1.**
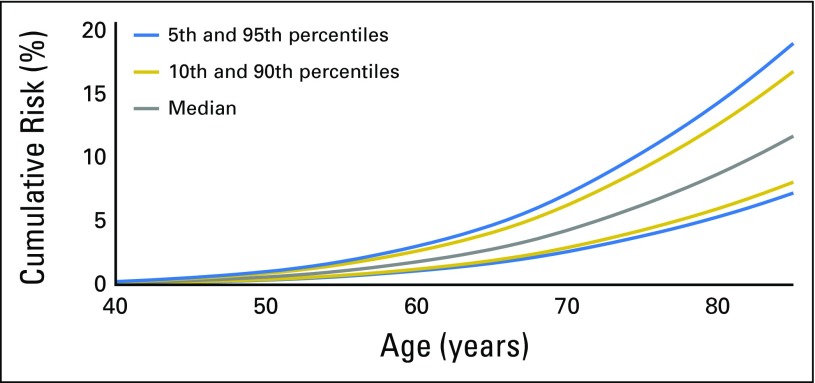
Predicted breast cancer cumulative risk for male carriers of *BRCA2* mutations by percentile of overall polygenic risk score that was constructed by using results from population-based studies.

**Fig 2.**
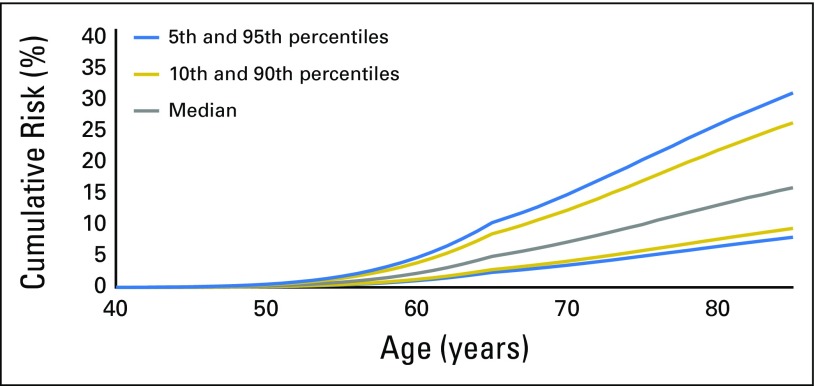
Predicted prostate cancer cumulative risk for male carriers of *BRCA1* mutations by percentiles of prostate cancer polygenic risk score that was constructed by using results from population-based studies.

**Fig 3.**
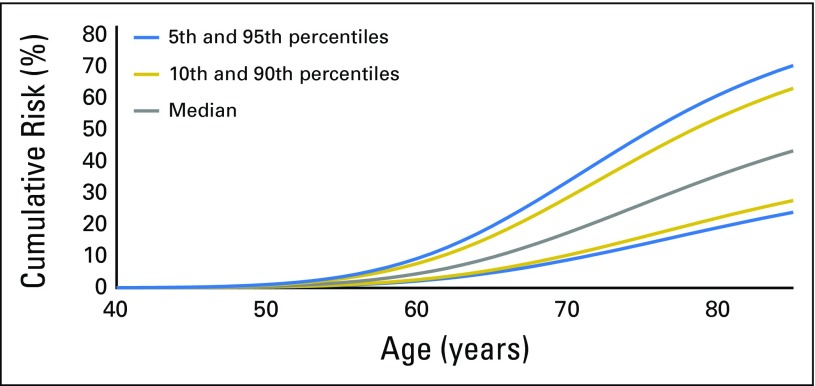
Predicted prostate cancer cumulative risk for male carriers of *BRCA2* mutations by percentiles of prostate cancer polygenic risk score that was constructed by using results from population-based studies.

We used the estimated OR for the breast cancer overall PRS and the prostate cancer PRS from the combined analysis of *BRCA1/2* samples to calculate male breast and prostate cancer risks at the 5th, 10th, 50th, 90th, and 95th percentiles of PRS distributions (Figs 1, 2, and 3 and Data Supplement). There were large differences in absolute risks between percentile groups. For *BRCA2* carriers, the risk of breast cancer by age 80 years is 5% for men at the 5th percentile of the PRS and 14% for men at the 95th percentile; the risk of prostate cancer by age 80 years is 19% for men at the 5th percentile of the PRS and 61% for men at the 95th percentile. For carriers of *BRCA1* mutations, men at the 5th percentile of the prostate cancer PRS have a 7% risk of developing prostate cancer by age 80, and men at the 95th percentile of the PRS distribution have a prostate cancer risk of 26%.

## DISCUSSION

We performed the first GWAS, to our knowledge, in male carriers of *BRCA1*/*2* mutations to identify common variants that modify the risks of breast and prostate cancer in these men. Although we analyzed the largest series of male mutation carriers available, this study is underpowered to detect associations with individual low-risk SNPs.

We have demonstrated that the combined effects of known breast cancer susceptibility SNPs modify breast cancer risk for male mutation carriers and, separately, that the combined effects of known prostate cancer susceptibility SNPs modify prostate cancer risk for male mutation carriers.

PRSs that were constructed with SNPs for female breast cancer and prostate cancer in the general population are highly predictive of risk in male carriers of *BRCA1/2* mutations. These results provide the first direct evidence of overlap in the genetic susceptibility to female breast and prostate cancers in the general population as well as the modification of risks of male breast and prostate cancer in men with *BRCA1/2* mutations.

We estimated an OR for breast cancer of 1.36 per SD increase in the overall breast cancer PRS. No study in the general population has assessed this exact PRS yet, but Mavaddat et al^15^ estimated an OR for female breast cancer of 1.55 for a PRS based on a subset of SNPs in females. Although the present estimate in males is not significantly different from that observed in females, it is somewhat lower. A lower OR may be a result of certain breast cancer SNPs that were included in the PRS that are not associated with male breast cancer risk, or individual SNPs may have smaller ORs for male breast cancer than female breast cancer. Alternatively, the estimate of Mavaddat et al^15^ may be susceptible to some level of winner’s curse bias.

The prostate cancer PRS was associated with prostate cancer risk in male carriers of *BRCA1/2* mutations, with an OR of 1.56 per SD increase in PRS. A previous study on prostate cancer PRS in the general population estimated an OR of 1.74.^14^

Overall, our results indicate that population-based breast and prostate cancer PRSs are predictive of cancer risk for male mutation carriers, which suggests a general model of susceptibility under which *BRCA1/2* mutations and other common cancer susceptibility variants interact multiplicatively on the risk of developing breast and prostate cancers.

To calculate PRSs we have used SNPs and corresponding log-OR estimates from external, population-based studies; therefore, the present analysis represents an independent validation of those externally derived PRSs and indicates that they are independently predictive of cancer risks for male carriers of *BRCA1/2* mutations. Although the present analysis was based on a case-control study design, information on SNPs is not subject to the usual biases that are associated with retrospective studies (eg, recall biases); therefore, the reported associations between the PRSs investigated and cancer risks are unlikely to be influenced by the study design.

The ER-positive PRS had a stronger association with male breast cancer in *BRCA1/2* mutation carriers than did the ER-negative PRS, which was in line with the observation that the majority of male patients with breast cancer among *BRCA1/2* mutation carriers are ER positive.^23^

We observed large differences in absolute risk between men in the bottom and the top of the PRS distribution. In particular, prostate cancer risk by age 80 years for male carriers of *BRCA1* mutations ranges from 7% for those at the bottom 5% of the risk distribution to 26% for those at the top 5% of the PRS distribution. By age 80 years, male carriers of *BRCA2* mutations are predicted to have a risk of prostate cancer that ranges from 19% for those at the bottom 5% of the risk distribution to 61% for those at the top 5% of the distribution, and a breast cancer risk that ranges from 5% to 14%.

In these calculations, we assumed conservative average prostate cancer risks for both *BRCA1* and *BRCA2* mutations; however, higher estimates for the effect of *BRCA1/2* mutations have been reported in the literature.^4,9^ Prospective studies of male mutation carriers will be useful for assessing the calibration of absolute cancer risks by PRS percentiles; however, such studies are not currently available with sufficiently large numbers of incident male breast and prostate cancer cases.

Although there are no established screening or intervention strategies for male carriers of *BRCA1/2* mutations, few clinical management recommendations include education, clinical breast examination, and prostate cancer screening.^39^ The present findings may inform the development of clinical recommendations on the basis of polygenic risk stratification of male mutation carriers to personalize management recommendations. For example, the current United Kingdom NICE guidelines recommend enhanced surveillance for women with a lifetime risk greater than 17% of developing breast cancer, regardless of their *BRCA1/2* status.^40^ Similar approaches may be developed for male carriers of *BRCA1/2* mutations for whom management would differ on the basis of their individual lifetime risk. For example, on the basis of the prostate cancer PRS, 43% of men with *BRCA1* mutations are predicted to have a prostate cancer risk of greater than 17% and may benefit from enhanced screening, whereas those at lower risk may opt for more limited surveillance.

Our data provide a strong impetus for new prospective screening studies in high-risk cohorts, such as the IMPACT trial,^41^ to include genetic risk assessment by PRSs in study protocols to assess the impact of cancer risk stratification in male mutation carriers. Recently, it has been suggested that polygenic risk-stratified screening can reduce overdiagnosis in the general population.^42-44^ Similar arguments may apply to male mutation carriers in whom polygenic risk prediction may further improve the effectiveness of screening.

A potential limitation of the current study is that family history information was not readily available for mutation carriers; therefore it was not possible to assess how the prostate and breast cancer risks in male carriers that are associated with PRSs vary by family history. Although this would not invalidate the association results, considering the effect of family history will be important in the context of genetic counseling.

Men with *BRCA1*/*2* mutations represent a small but unique patient group in terms of clinical management. Our results suggest that risk profiling on the basis of PRSs may identify male carriers of *BRCA1*/*2* mutations at both sufficiently reduced or increased risk of breast or prostate cancer, with implications for their clinical management. To facilitate this, it will be important to incorporate such PRSs into breast or prostate cancer risk prediction algorithms.^45^

As an accurate risk assessment is the basis of cancer prevention and screening strategies, the PRSs presented here may be used to provide male carriers of *BRCA1/2* mutations and their physicians with more detailed information on their breast and prostate cancer risks to aid prevention and screening decisions.
